# A 107 Gene Nanostring Assay Effectively Translates the Cancer Genome Atlas, and Tumour Microenvironment Gastric Cancer Molecular Classification to a Patient‐Derived Organoid Model

**DOI:** 10.1002/gcc.70090

**Published:** 2025-11-12

**Authors:** D. Skubleny, K. Purich, T. Williams, D. R. McLean, S. N. Martins‐Filho, K. Buttenschoen, E. Haase, M. McCall, K. Baker, S. Ghosh, J. L. Spratlin, D. E. Schiller, G. R. Rayat

**Affiliations:** ^1^ Department of Surgery Faculty of Medicine and Dentistry, University of Alberta Edmonton Alberta Canada; ^2^ Department of Laboratory Medicine and Pathology University of Alberta Edmonton Alberta Canada; ^3^ Department of Oncology Faculty of Medicine and Dentistry, University of Alberta Edmonton Alberta Canada; ^4^ Department of Mathematical and Statistical Sciences Faculty of Science, University of Alberta Edmonton Alberta Canada

**Keywords:** gastric cancer, molecular classification, nanostring, organoid, tumour immune microenvironment

## Abstract

There is a need to improve the translation of gastric cancer molecular classification schemes, such as those proposed by the Cancer Genome Atlas (TCGA) and Tumour Microenvironment score (TME), to clinical specimens and three‐dimensional organoid culture models. In this study, we validate a 107‐gene Nanostring assay informed by previously established machine learning models using a prospective cohort of gastric adenocarcinoma tumours and tumour‐organoid pairs. Thirty‐eight gastric adenocarcinoma specimens and twelve parent tumour‐tumour organoid pairs were assigned TCGA and TME subtypes using gene expression measured by our custom Nanostring gene set. Subtypes were validated using gold‐standard tests for Epstein–Barr virus (EBV) and microsatellite instability (MSI). Molecular subtype scores were compared to known clinicopathologic characteristics. The correlation between dose–response and molecular subtypes using an organoid drug assay and the Cancer Cell Line Encyclopedia (CCLE) was investigated. TCGA and TME subtypes were successfully applied to all specimens. The relationship of molecular subtype scores in our population compared to public cohorts was statistically identical for Lauren Class and Signet Ring status. Our method achieved 100% accuracy in labeling EBV and MSI subtypes. We identified 81.8% and 63.6% concordance between parent tumour‐tumour organoid pairs for TME and TCGA subtypes, respectively. No significant correlation was identified between dose response to chemotherapy and molecular subtype scores. Analysis of the CCLE identified promising personalized therapy candidates for each molecular subtype. Our 107‐gene Nanostring test successfully assigns TCGA and TME molecular subtypes to clinical tumour and tumour organoid samples for use in future study.

**Abbreviations**
ACRG: Asian cancer research group
CCLE: cancer cell line encyclopedia
CIN: chromosomal instability
DSS: drug sensitivity score
EBER ISH: Epstein–Barr encoded early RNAs in situ hybridization
EBV: Epstein–Barr virus
FLOT: 5‐fluorouracil, leucovorin, oxaliplatin and docetaxel
FSQN: feature specific quantile normalization
GS: genomically stable
MSI: microsatellite instability
RUV‐III: removal of unwanted variation–III algorithm
TCGA: the cancer genome atlas
TME: tumour microenvironment score

## 1 Introduction

The development of personalized medicine for gastric adenocarcinoma is an intriguing concept given the significant molecular heterogeneity of this disease. Furthermore, clinical trial failure for targeted therapy is a common occurrence in gastric cancer [1]. Patient‐derived organoids and molecular classification are promising methods to advance personalized medicine in gastric cancer. Three‐dimensional (3D) cell culture to create “mini‐organs” called organoids may be used to evaluate molecular cancer biology, assess pre‐clinical therapeutic efficacy or potentially guide personalized treatment [2, 3, 4]. As such, some have suggested that 2‐dimensional (2D) cell culture models could be displaced by organoids [5]. Several molecular classification systems have provided insight into the molecular heterogeneity of gastric cancer including those proposed by the Cancer Genome Atlas (TCGA) classification, Asian Cancer Research Group (ACRG) and the Tumour Microenvironmental score (TME) by Zeng et al. among others [6, 7, 8, 9]. These novel classification methods inform gastric cancer prognosis. However, their clinical utility and impact on therapeutic decision‐making is lacking.

Recent trials have established immunotherapy as a viable treatment option for gastroesophageal junction cancer and metastatic gastric cancer in the adjuvant and palliative settings, respectively [10, 11]. In the neoadjuvant setting, there is a need to identify favorable tumour populations given that only 40% of patients achieve complete or partial treatment response to cytotoxic chemotherapy [12, 13, 14]. Clinical trials investigating the efficacy of immunotherapy in the neoadjuvant setting are still ongoing [15, 16]. The expansion of molecular classification to investigate subtypes such as microsatellite instability (MSI) or Epstein–Barr virus type (EBV) tumours, as defined by the TCGA classification, has shown promise. For example, data has demonstrated that PD‐1 checkpoint inhibition therapy such as pembrolizumab in MSI and EBV tumours is efficacious [17]. The utility of cytotoxic neoadjuvant chemotherapy regimens such as FLOT or MAGIC in MSI tumours has also been a significant debate [18, 19, 20, 21, 22]. An improved understanding of molecular classification in gastric cancer is required to identify novel therapeutic options and enhance contemporary management.

We recently developed a set of supervised machine learning classifiers to perform an integrated analysis of the TCGA, ACRG and TME molecular classification systems in gastric adenocarcinoma [23]. This analysis identified the TME score as the most relevant prognostic signature that may also inform therapeutic response. To enable the application of these molecular classification systems to future translational research we sought to develop, validate and apply a 107‐gene Nanostring CodeSet informed by our previously established machine learning classifiers to a prospective patient cohort and patient‐derived 3D organoid cultures.

## 2 Methods

### 2.1 Prospective Cohort Study Design

**FIGURE 1:**
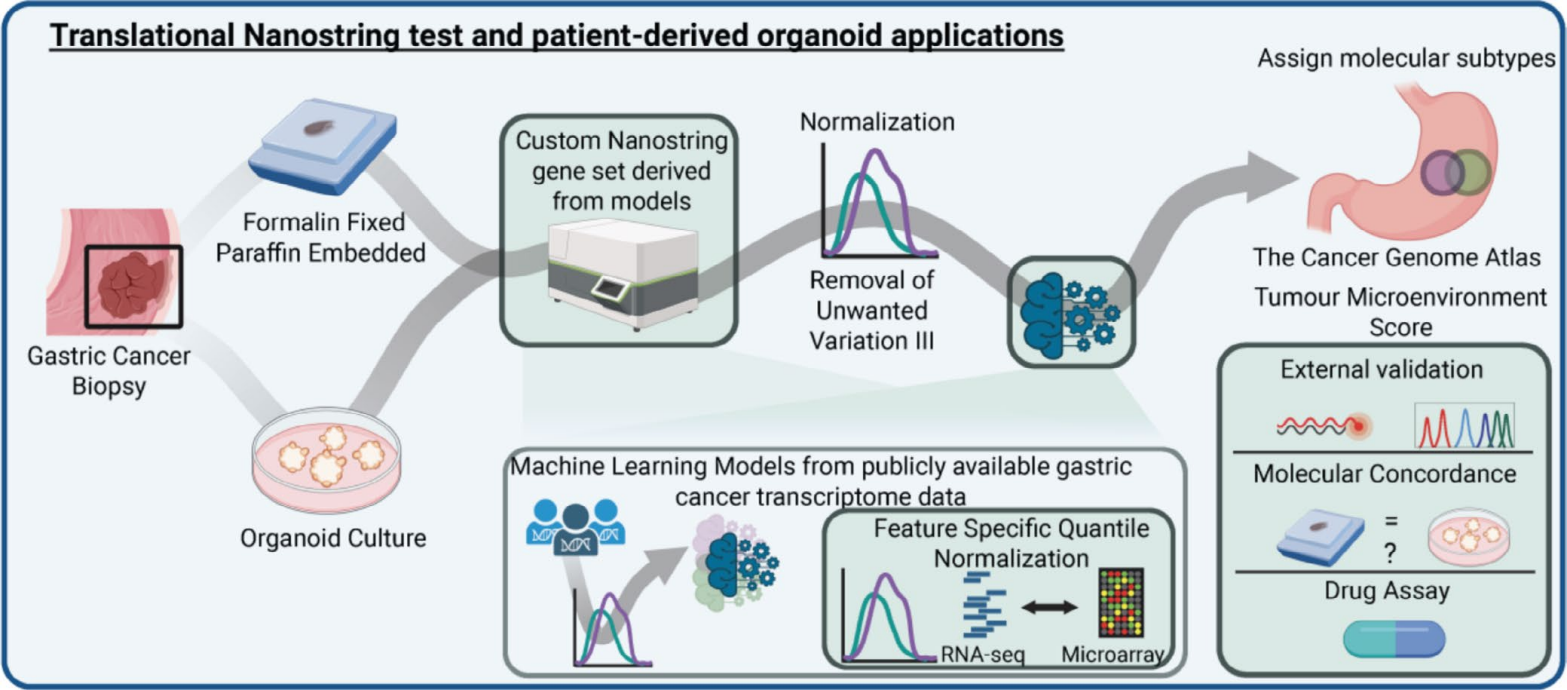
Study overview. Biopsies of gastric cancer were formalin‐fixed paraffin‐embedded (*n* = 38) and established as organoid culture (*n* = 12). Nucleic acids were isolated and gene expression was measured using a 107‐gene Nanostring codeset. Gene expression data was normalized and used to assign molecular subtypes to gastric cancer samples using previously characterized machine learning models. Molecular subtypes were then externally validated and the molecular concordance between tumour and tumour organoid pairs was analyzed. An in vitro organoid drug assay was performed. Image created with BioRender.com.

We performed a single‐center, prospective study at the University of Alberta in Edmonton, Alberta, Canada from June 2019 to January 2022. All human clinical participants consented according to the approved ethics protocol granted by the Health Research Ethics Board of Alberta (Study ID: HREBA.CC‐17‐0228). Treatment naïve Stage I–IV sporadic gastric adenocarcinoma patients older than 18 years were included. Patients with a known inherited oncogenic germline mutation or hereditary syndrome (i.e., Familial Adenomatous Polyposis) were excluded. The study overview is illustrated in Figure 1A.

Specimens were retrieved via endoscopic biopsy at the time of diagnosis, staging laparoscopy or surgical resection at the Walter C. Mackenzie Health Sciences Centre or Royal Alexandra Hospital. Four biopsies were collected for permanent pathology in Z‐fix (Sigma) for cancer and adjacent normal tissue, respectively. Four additional cancer and adjacent normal biopsies were collected to generate patient‐derived organoids.

### 2.2 Organoid Culture

We generated human gastric organoids according to our previously described intestinal organoid protocol [24]. Specific reagent information and catalogue numbers can be found in Table S1. Briefly, gastric cancer biopsies were minced and subsequently dissociated in 20 mL digestion buffer containing Advanced DMEM/F12, 100 units/mL penicillin and 100 μg/mL streptomycin, 100 μg/mL Primocin, 2.5% FBS, 75 units/mL Collagenase XI and 125 μg/mL Dispase II and placed in a mechanical water bath at 37°C for 1 h. Cells were resuspended in ice‐cold 70% Matrigel (Corning, 356253) in Advanced DMEM/F12 at a concentration of 1000 cells/μL and 35 μL Matrigel domes were placed in a prewarmed 24 well tissue culture treated plate. Organoids were cultured in 500 μL organoid culture medium (see Supporting Information) at 37°C and 5% CO_2_ until mature for passaging or downstream analysis [25].

Normal gastric organoids were passaged approximately every 7–14 days. Cancer organoids were passaged every 7–21 days due to cancer‐specific variable growth rates. Organoids were dissociated using TrypLE Express (Gibco, 12604013) and replated in 35 μL Matrigel domes as above. Dissociated organoid cells were further cultured or allocated for molecular analysis or dose–response drug assays.

### 2.3 Nanostring Assay

We designed a custom Nanostring CodeSet using the genes selected in our machine‐learning models. The list of genes used for our custom Nanostring CodeSet can be found in our GitHub Repository (https://github.com/skubleny/Integrated‐Molecular‐Classification‐GC). The TCGA and TME classification systems were chosen to evaluate the utility of our proposed method. Nucleic acids from organoids and FFPE curls were isolated using the AllPrep DNA/RNA kit (Qiagen, 80204) and AllPrep FFPE DNA/RNA kit (Qiagen, 80234), respectively. Details for isolation techniques are provided in the Supporting Information. Measurement of gene expression using our custom CodeSet was performed by the Laboratory Medicine and Pathology core. Overnight hybridization of 100 ng of total RNA was performed followed by measurement of gene counts using the Nanostring nCounter machine.

Normalization of Nanostring data was performed according to Molania et al. [26] Raw Nanostring counts were imported to nSolver software 4.0 and samples with quality control flags were excluded. All included samples were subsequently normalized using the Removal of unwanted variation‐III algorithm (RUV‐III) with technical replicates spanning different Nanostring cartridges [27, 28]. Following RUV‐III normalization, Feature Specific Quantile Normalization (FSQN) was used to transform gene expression values to the target distribution of TCGA and TME classifiers [29, 30]. Molecular subtypes were learned using previously characterized models [23]. For technical replicates, we used the mean value of subtype scores.

### 2.4 Over‐Representation Analysis

The relationship of the TME score genes to molecular gene sets was performed in R using clusterprofiler [31]. Over representation analysis was performed on the Hallmark gene collection and Gene Ontology (GO) gene collections for Molecular Function (MF), Biological Process (BP) and Cellular Component (CC) from the Molecular Signature Database [32, 33, 34, 35]. Significant gene signatures were selected with adjusted *p* values < 0.05 following Benjamini‐Hochberg correction. The results were visualized in a social network plot using igraph and ggraph [36, 37].

### 2.5 Immunofluorescence and Immunohistochemistry

Tissue biopsies and organoids were processed as previously described [24]. Specific antibody combinations, dilutions, incubation times and antigen retrieval buffers are listed in Table S2. Microwave heat‐induced antigen retrieval was performed using sodium citrate (pH 6, heated to 94°C in 1‐min intervals followed by 9 min of continuous heat) or Tris‐EDTA (pH 9, heated to 94°C in 1‐min intervals followed by continuous heat for 8 min 30 s).

For immunofluorescence, permeabilization was performed with 0.5% Triton X‐100. Non‐specific epitopes were blocked using 10% normal goat serum. Tissue sections were stained with primary antibodies (mouse anti‐pan cytokeratin, 1:25, rabbit anti‐vimentin, 1:300) and secondary antibodies (goat anti‐mouse IgG Alexa Fluor 568, 1:200, Invitrogen, A‐11004 and goat anti‐rabbit IgG Alexa Fluor 488, 1:200, Abcam, ab150077). Autofluorescence was diminished using a TrueView Quenching kit per the manufacturer's protocol (Vector, SP‐8400‐15). Nuclear counterstaining was performed with DAPI followed by coverslipping with Vectashield Vibrance Antifade mounting media (Vector, H‐1700). Images were captured on an AxioCam HRc camera and processed using ImageJ [38].

For immunohistochemistry, endogenous peroxidases were quenched with 3% hydrogen peroxide in methanol. Non‐specific binding was mediated by blocking with 20% normal goat serum and avidin/biotin blocker per the manufacturer's protocol (Vector Laboratories, 2001). Mouse anti‐MUC5AC primary antibody or rabbit anti‐pepsinogen II/PGC was diluted 1:250 and 1:100, and incubated at 4 degrees Celsius overnight, respectively. Goat anti‐rabbit or goat anti‐mouse biotinylated IgG secondary antibodies were incubated at 1:200 dilution for 30 min for anti‐PGC and anti‐MUC5AC, respectively. Antibody detection was performed using avidin‐biotin complex/horseradish peroxidase (Vector Laboratories) and 3,3‐diaminobenzidine tetrahydrochloride (DAB, Abcam, ab64238) per the manufacturer's protocol. Tissues were counterstained with hematoxylin. Brightfield microscopy images were captured using a Leica Aperio CS2 digital slide scanner.

### 2.6 Epstein–Barr Encoded Early RNAs In Situ Hybridization

To confirm that our Nanostring‐derived TCGA EBV score captured true EBV‐positive tumours we used gold‐standard Epstein–Barr encoded early RNAs in situ hybridization (EBER ISH) in parent tumour and organoids [39]. Detection of EBER ISH was performed on FFPE tissue sections using biotinylated alkaline phosphatase mediated in situ hybridization with the Rembrandt detection kit according to the manufacturer's protocol (ThermoFisher, A500K.0105). Tissue sections were counterstained using nuclear fast red.

### 2.7 Microsatellite Instability

Pentaplex polymerase chain reaction (PCR) is the gold‐standard method for assessing tumour microsatellite instability (MSI) [40, 41]. DNA was isolated from FFPE sections of tissues and organoids in paired normal and cancer tissue as above. The concentration of mRNA‐free genomic DNA was quantitatively measured using QuBit (ThermoFisher). MSI status was tested using the MSI Analysis System, Version 1.2 (Promega, MD1641). Briefly, 2 ng of genomic DNA was amplified using GoTaq MDx Hot Start polymerase (Promega, D6001) on a BioRad T100 thermal cycler per manufacturer‐recommended settings. The Applied Genomics Core at the University of Alberta performed the capillary electrophoresis on an Applied Biosystems 3130XL Genetic Analyzer. Data was processed using Geneious Prime version 2021.2.2. Alleles were called using 2nd order least squares. Samples were deemed MSI‐High, MSI‐Low, or microsatellite stable if allelic variations were found in ≥ 2, 1 or 0 microsatellite markers, respectfully.

### 2.8 In Vitro Dose Response Drug Assay

Our combination FLOT (5‐fluorouracil, leucovorin, oxaliplatin and docetaxel) dose–response assay was validated using the AGS (RRID:CVCL_0139) human gastric cancer cell line (ATCC CRL‐1739). Details for AGS culture, drug assay validation and dose response analysis are provided in the Supporting Information. Organoids were dissociated and 5000 cells were plated in 96‐well plates and grown in organoid media for 24 h followed by 48 h of FLOT treatment and viability assessment using a CCK‐8 assay. Anti‐cancer drugs 5‐fluorouracil (Tocris, 3257), oxaliplatin (Tocris, 2623) and docetaxel (Tocris, 4056) were added in triplicate over 8 half‐log dilutions (5‐fluororacil and oxaliplatin) or 10‐fold dilutions (docetaxel). Initial concentrations for 5‐fluorouracil, oxaliplatin and docetaxel were 800 μM, 2400 μM and 2400 nM, respectively. A single 500 μM dose of leucovorin (Toronto Research, L330400) was added to each treatment well. Nonlinear regression of dose–response data was performed using GraphPad Prism version 9 to calculate half‐maximal inhibitory concentration (IC50) values. Drug Sensitivity Score (DSS) was calculated using estimated parameters from nonlinear regression using the DSS package [42].

### 2.9 Cancer Cell Line Encyclopedia Analysis

We retrieved multi‐omics data and dose–response data for gastric cancer cell lines present in the Cancer Cell Line Encyclopedia [43]. Data were downloaded from the Broad DepMap Portal (https://depmap.org/portal/download/). A complete list of datasets used for this analysis is available in the Supporting Information (https://github.com/skubleny/Integrated‐Molecular‐Classification‐GC).

RNA‐seq data were retrieved as log_2_ transformed RNA‐Seq by Expectation–Maximization (RSEM) counts [44]. Molecular subtypes for TCGA, ACRG, and TME classification were assigned using our previously characterized supervised classifier method [23]. Area under the curve drug sensitivity values were retrieved from the Sanger Genomics of Drug Sensitivity in Cancer (Sanger GDSC2) [45]. We filtered out drugs with greater than 25% missing data. Pearson's correlation of subtype score and drug sensitivity was performed. Pearson's correlation values were then pooled according to drug targets as specified by GDSC. A Kruskal‐Wallis test evaluated drug target categories for Pearson's correlation of drug efficacy and molecular subtype score.

## 3 Results

### 3.1 Organoid Model

**FIGURE 2:**
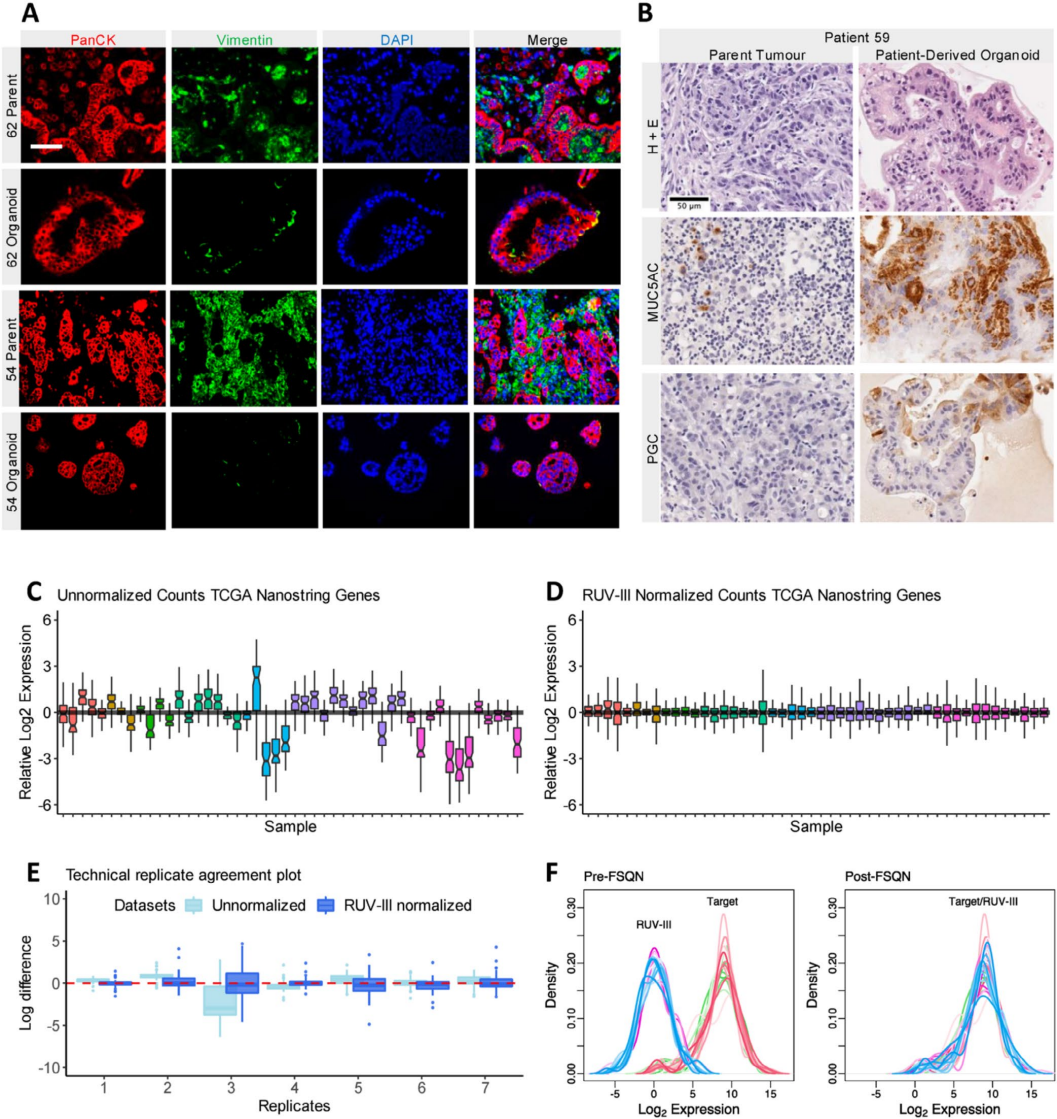
Representative images of human gastric cancers and patient‐derived organoids, Nanostring normalization and feature specific quantile normalization, (A) Immunofluorescence demonstrating recapitulation of stromal cells within organoids. Pan cytokeratin (red) is present on epithelial cell membranes, Vimentin (green) is a stromal cell specific marker and DAPI (blue) was used to stain nuclei. (B) Tumours and patient‐derived organoids demonstrated similar morphology on hematoxylin and eosin stains. The gastric specific markers MUC5AC and PGC were present in both parent and organoid tissues. Scale bar represents 50 μm. (C) Boxplots for 39 formalin‐fixed paraffin‐embedded specimens demonstrating the distribution of unnormalized log_2_ expression for 57 TCGA genes. Boxplot notches approximate the 95% confidence interval of the median. (D) Boxplots showing the same samples from (C) following RUV‐III normalization. (E) Boxplot showing comparison of unnormalized and normalized log difference between 7 samples that we used as technical replicates for RUV‐III normalization of patients in (C, D). Optimal technical agreement should approach zero and improvement relative to unnormalized samples should be observed. (F) Example of Feature Specific Quantile Normalization (FSQN) for same patients from (C–E). FSQN normalizes the RUV‐III distribution developed using our Nanostring assay to the target distribution of the TCGA RNA‐seq data used to train our machine learning classifier.

To externally validate the custom Nanostring CodeSet we prospectively enrolled 38 gastric cancer patients, of which patient‐derived tumour organoids were developed in a subset of 12 patients. Two pathologists (D.R.M, S.N.M‐F) assessed parent tissue biopsies to confirm the presence of cancer. Our success rate of establishing organoid culture was 75%, with two samples failing to grow and two others experiencing fungal contamination. Histology of parent tumour and tumour‐organoid pairs largely revealed consistent tissue morphology. One parent tumour (patient 67) did not contain cancer; thus the paired organoid was removed from subsequent drug assay analyses due to the functional relevance of cancer specific tissue. However, the malignancy status did not preclude its inclusion in the EBV ISH and MSI pentaplex PCR validation. In Figure 2A representative images demonstrate morphological recapitulation of the parent tumour in patient‐derived organoids.

To ensure recapitulation of gastric epithelium we assessed the expression of PGC and MUC5AC in parent tissue and patient‐derived organoids (Figure 2B). Concordant positive staining of at least one of MUC5AC or PGC was observed between all tumour‐organoid pairs. The presence of epithelial and stromal tissue was confirmed using immunofluorescent staining of pan‐cytokeratin and vimentin. Minimal vimentin expression was observed in diffuse‐type organoids (Patient 54) relative to differentiated intestinal‐like organoids (Patient 62).

### 3.2 Normalization Technique

Normalization according to methods proposed by Molania et al. provided reliable elimination of sample and batch differences (Figure 2C,D) [26]. We observed a close approximation of technical replicates following normalization with the median log differences values approaching zero (Figure 2E). FSQN provided an excellent approximation of normalized Nanostring gene expression data to target distributions to learn molecular subtypes from our previously established supervised machine learning model (Figure 2F).

### 3.3 Our Nanostring Assay Is Externally Valid for Gold Standard Features

**FIGURE 3:**
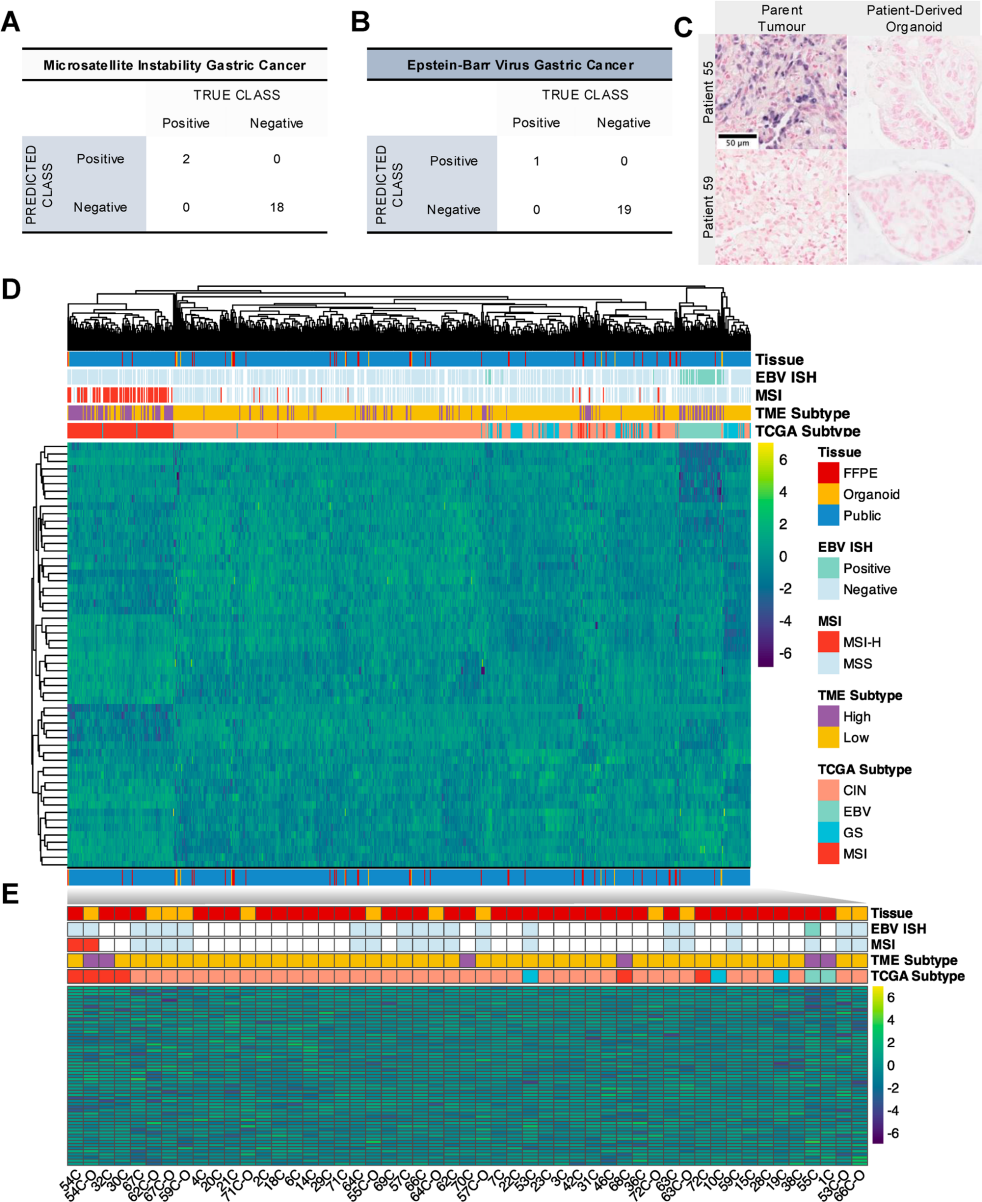
Validation of our Nanostring test in a clinical cohort and patient‐derived organoids. (A) Contingency table comparing allocation of Epstein–Barr Virus (EBV) subtypes using our test versus the gold‐standard EBER ISH. (B) Contingency table comparing allocation of Microsatellite Instability (MSI) subtypes using our test versus the gold‐standard pentaplex PCR. (C) Representative images of EBER ISH in two tumour organoid pairs. Parent tumours are shown in the left column and the patient‐derived organoid is shown in the right. (D) Heatmap plot of gene expression values for 57 TCGA genes. Genes are rows and patients are columns. Rows and columns are clustered using complete linkage based on Euclidean distances of scaled gene expression values. The origin of the tissue sample, the assigned TCGA and TME subtype according to our machine learning model and the independent MSI and EBV ISH status are illustrated in the annotation legend above the plot. Gene expression value corresponds to the continuous legend in the top right. (E) Sub‐graph heatmap derived from Figure 3D containing only the Edmonton cohort patients. Patient order is preserved from the clustering in the heatmap above.

We externally validated our Nanostring CodeSet using our cohort of patient‐derived organoids and parent tissue samples, with reference to gold‐standard tests for EBV and MSI tumours. We performed EBV ISH and pentaplex PCR on 10 paired parent and organoid samples. In these samples, our Nanostring test was 100% accurate in assigning EBV and MSI classes (Figure 3A,B). Figure 3C illustrates a positive and negative EBV ISH test in parent tumour samples (top left and bottom left, respectively) and their corresponding organoid (top and bottom right).

We compared the Nanosting gene expression values from the 57 TCGA classifier genes measured on our samples within the context of the TCGA and ACRG cohorts. We chose these cohorts because they also provided independent EBV ISH and MSI status. The scaled gene expression values are presented in the heatmap in Figure 3D. Distinct clusters were identified including dominant clusters for MSI and EBV tumours. Importantly, the MSI and EBV status derived from our machine learning models also corresponded to the MSI and EBV status derived from the independent PCR and EBV ISH tests. Our organoid and FFPE samples were distributed throughout the cluster dendrogram in subtype‐appropriate locations suggesting accurate representation of the TCGA gene expression from our Nanosting test.

Figure 3E is a direct subgraph of our cohort samples. We identified reasonable distances between some parent‐organoid samples, such as patient 57, 63, 64, 67, 71 and 72. Patient 54C and organoid 54C‐O clustered together within the MSI dominated clade. Discordance between organoid and parent tissue in patient 53, 55 and 66 was also identified.

### 3.4 Selected TME Genes Reveal Interactions Between Stromal and Immune Pathways

**FIGURE 4:**
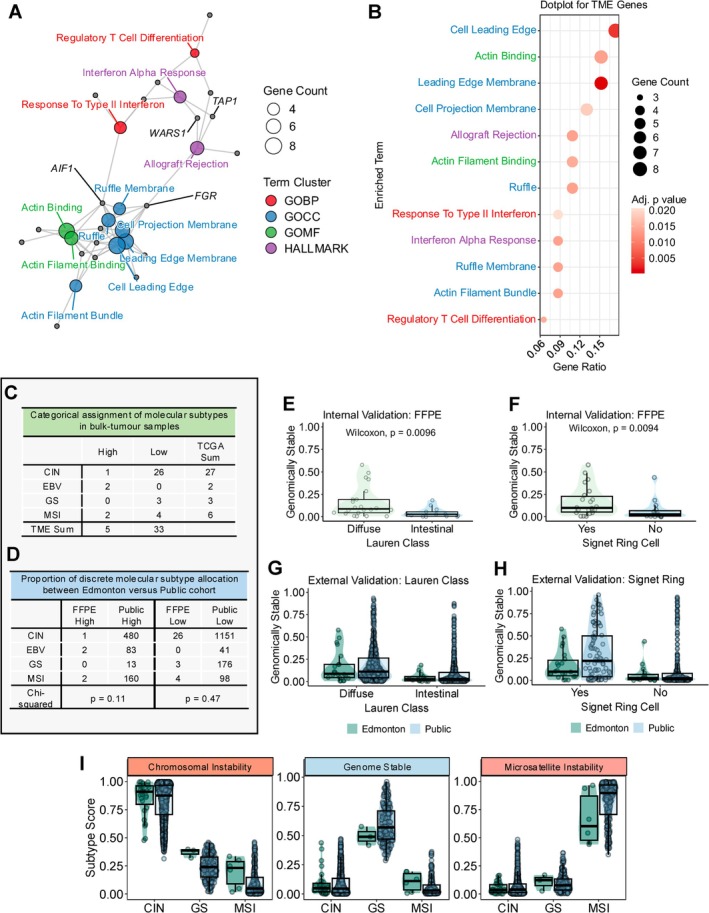
Validation of our Nanostring test in a clinical cohort and patient‐derived organoids. (A) Gene network plot for top 15 statistically significant molecular signature terms from over‐representation analysis of 50 TME score genes. The force‐directed graph layout is informed by the Fruchterman‐Reingold algorithm. Each coloured node represents a gene signature corresponding to the size and collection denoted by the figure legend. Grey nodes represent individual genes. Relevant hub‐genes are labeled. (B) Dotplot of top 15 statistically significant enriched molecular signature terms from over‐representation analysis of 50 TME score genes. Gene ratio of identified genes is presented on the x‐axis and the dot size corresponds to the total gene count. Molecular signature terms are presented on the y‐axis. The dot color corresponds to the adjusted *p* value following Benjamini‐Hochberg correction. (C) Assignment of TCGA and TME molecular subtypes in 38 bulk tumour samples using the custom Nanostring codeset informed by our supervised classification models. (D) Assignment of TCGA molecular subtypes stratified by TME status in 38 bulk tumour samples using the custom Nanostring codeset informed by our supervised classification models compared to the assignment of molecular subtypes in 2202 publicly available patients classified according to our supervised classification models. A Chi‐Square test was used to assess for difference in proportions. (E) Boxplot of genomically stable scores in 38 formalin‐fixed paraffin‐embedded samples versus Lauren classification. (F) Boxplot of genomically stable scores in 38 formalin‐fixed paraffin‐embedded samples versus signet ring cell status. (G) Boxplot of genomically stable scores versus Lauren classification status in 38 formalin‐fixed paraffin‐embedded samples compared to the public cohort of 2202 patients. (H) Boxplot of genomically stable scores versus signet ring cell status in 38 formalin‐fixed paraffin‐embedded samples compared to the public cohort of 2202 patients. (I) Distribution of molecular subtype scores for each molecular subtype stratified by the discrete molecular class allocation. Results are shown for molecular classes with ≥ 3 assigned samples.

The social network derived from the top 15 statistically significant gene sets for TME score genes is presented in Figure 4A. Gene‐ratios and the accompanying Benjamini‐Hochberg adjusted *p* values are provided in Figure 4B. Over‐representation analysis identified stromal and cellular features consistent with the tumour microenvironment from GO:CC and GO:MF collections. Gene sets were enriched in actin binding, leading edge membrane and ruffle membrane functions. We also identified enrichment in Hallmarks Interferon Alpha Response and Allograft Rejection, as well as GO:BP Regulatory T Cell Differentiation and Response to Type II Interferon. Relevant hub genes amongst the immune gene sets included WARS1 and TAP1. Two prominent hub‐genes (AIF1 and FGR) were identified between the immune activation and stromal/cellular motility‐based gene sets.

### 3.5 Demographics of the Prospective Cohort and Evaluation of Molecular Subtype Assignment in Bulk Tumour Samples

Demographic data for 38 patients in our prospective cohort are found in Table S3. The median age was 65 (IQR 60, 74) with a predominance of male patients (67%). All stages were equally present, but most tumours were poorly differentiated (G3 = 68%). Most tumours were also proximal, diffuse and exhibited signet ring cell features.

Following appropriate normalization procedures, we used our previously characterized supervised classifiers to assign TCGA and TME classes and scores. The assigned molecular subtypes of our patient population using Nanostring gene counts measured from FFPE specimens are provided in Figure 4C. The proportion of TCGA classes was largely concordant with the subtypes assigned in the public cohort of 2202 patients (Figure 4D). Chromosomal instability was observed in 71.1% of patients (Public cohort = 74.1%), EBV in 5.3% (Public cohort = 5.6%), GS in 7.9% (Public cohort = 8.6%) and MSI in 15.8% (Public cohort = 11.7%). However, the prevalence of TME High tumours was less in our population (13.2% vs. 33.4%). Among TME High tumours we observed the expected presence of EBV‐ and MSI‐TME High classes but the prevalence of CIN‐TME High tumours was lower than the reference population (3.7% vs. 21.8%). In Figure 4D we identified that the distribution of TCGA subtypes in both TME High and Low tumours between our cohort and the public data did not reach statistical significance (Chi‐Square tests *p* > 0.05).

Given that we did not perform whole genome sequencing in this preliminary study, we assessed the validity of GS assignments using pathology characteristics. Genomically stable tumours have previously been demonstrated to be enriched in diffuse‐type gastric cancer and signet ring cells [7, 46]. Our test recapitulated this relationship with significantly greater GS score in diffuse cancers relative to intestinal‐type (Figure 4E) (Wilcoxon test, *p* < 0.05). In Figure 4F we also confirmed greater GS scores in signet ring cell tumours (Wilcoxon, *p* < 0.01). We then externally validated the GS scores generated by our Nanostring test by comparing the distribution of GS scores between our Edmonton cohort and the public cohort of 2202 patients for both Lauren class and signet ring cell status (Figure 4G,H). No statistically significant differences were identified (Wilcoxon, *p* > 0.5). In Figure 4I we also demonstrate no significant differences among subtype scores between our FFPE samples and the public cohort when stratified by discrete molecular subtype allocations (Wilcoxon, *p* > 0.05). These analyses demonstrate that our Nanostring test accurately represents the expected distribution of scores concerning known pathologic variables and molecular scores.

### 3.6 Molecular Subtype Discordance Exists Between Parent Tumour and Tumour Organoid Pairs

**FIGURE 5:**
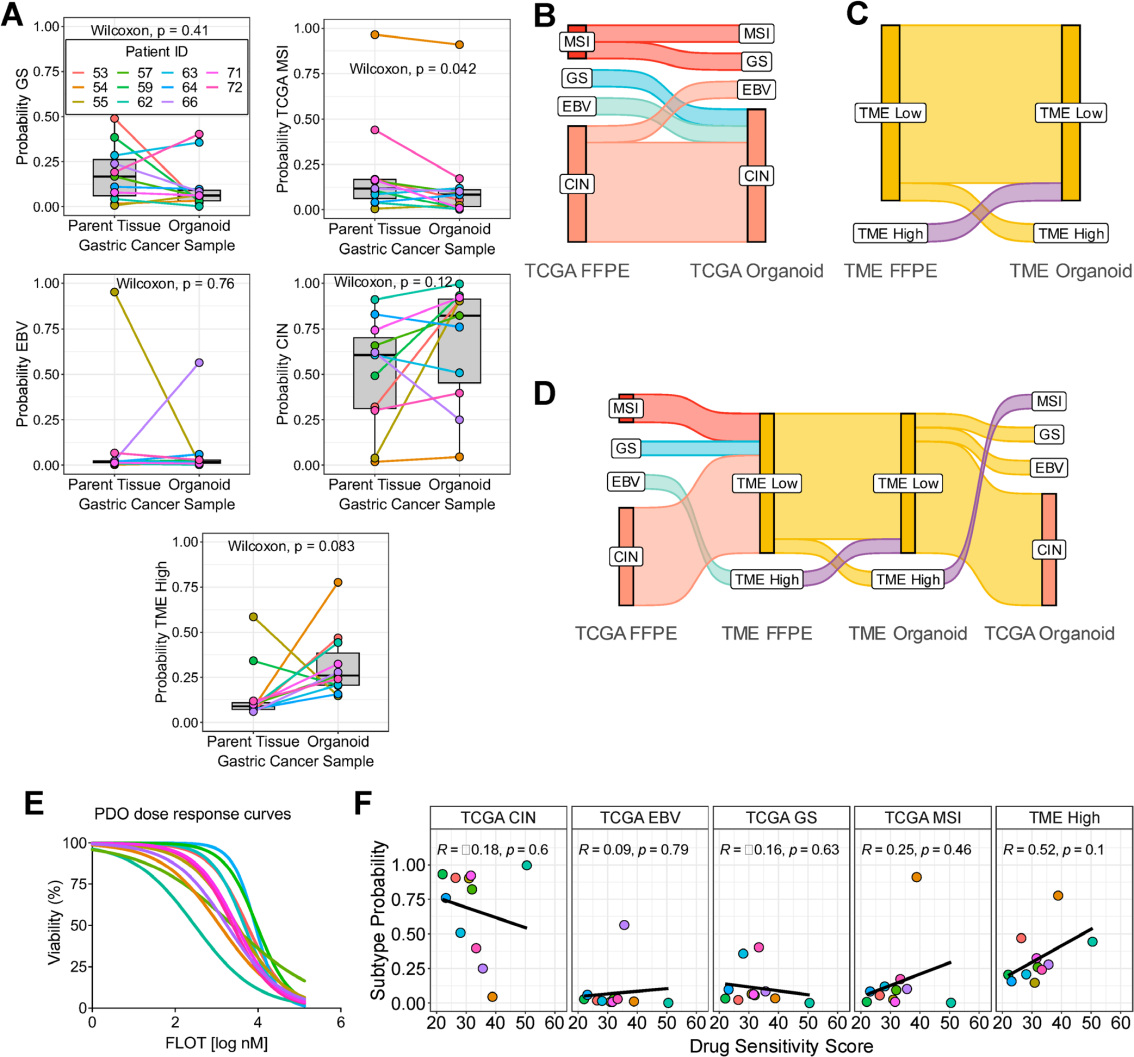
Assessment of concordance of molecular subtype scores between tumour organoid pairs and association of subtype scores with FLOT drug sensitivity. (A) Paired boxplots comparing concordance of molecular subtype scores between 11 parent tumours and patient‐derived organoid pairs (*x*‐axis). The subtype is designated by the plot title. The *y*‐axis represents the corresponding subtype score. *p* values for a paired Wilcoxon test are presented in each plot. (B) Sankey plot demonstrating the concordance between TCGA molecular classification between FFPE and organoid pairs. For (B–D), the *x*‐axis denotes the classification and specimen type. Each patient is represented in every column and molecular subtypes are denoted by colour and label. (C) Sankey plot demonstrating the concordance between TME molecular classification between FFPE and organoid pairs. (D) Sankey plot demonstrating the complete flow of TCGA and TME molecular subtypes for 11 patient‐organoid pairs from FFPE to organoid samples. (E) Dose response curves for 10 patient‐derived organoids (PDO) treated with FLOT chemotherapy in vitro. The *x*‐axis represents the log of FLOT concentration in nanomoles. Cell viability was measured using a CCK‐8 assay. (F) Scatterplots illustrating the association of molecular subtype probability versus drug sensitivity score (DSS) in 10 patient‐derived organoids for each molecular subtype as designated in the plot title. Spearman's rho coefficient and *p* value are represented in each plot. The black line represents the line of best fit using simple linear regression.

After establishing the validity of our test, we assessed the propensity of patient‐derived organoids to recapitulate the molecular subtype of their parent tumour sample. Using categorical assignment of subtypes, we found 81.8% and 63.6% concordance between parent tumours and tumour organoids for TME and TCGA classes, respectively (Chi‐Square tests *p* > 0.05) (Tables S4 and S5). A paired analysis of continuous molecular subtype scores found that MSI scores were significantly different between parent tumour and tumour organoids (Paired Wilcoxon, unadjusted *p* = 0.04) (Figure 5A).

We assessed the allocation of discrete molecular classes between parent tumour‐organoid pairs for TCGA and TME classification. Here, we found that parent tissue molecular classes derived from FFPE analysis were prone to transitioning to CIN subtypes within the organoid culture (Figure 5B). In Figure 5C, we demonstrate that most parent tumours successfully recapitulated the TME Low subtype in the organoid culture, but that subtype discordance did occur with two parent tumour‐organoid cultures. In general, the allocation of subtypes within FFPE samples followed expected trajectories (Figure 5D). For example, CIN FFPE samples were associated with TME Low tumours. Likewise, the only EBV tumour was TME High within FFPE tissues. Patient 54 generally followed an expected pattern of subtypes with an MSI‐High parent tissue and an MSI‐High/TME High organoid. However, we observe discordance of these patterns between some parent tissue and organoid pairs. For example, the EBV positive/TME High type tumour from patient 55 formed a TME Low/CIN dominated organoid characteristic.

### 3.7 Organoid Dose–Response Drug Assays

Dose–response drug assays using FLOT chemotherapy were completed for the 11 patient‐derived organoids with confirmed malignancy in the parent tissue. First, we successfully validated the reproducibility of our method using the AGS cancer cell line across three independent dose–response assays (Figure S1).

Least squares nonlinear regression was well fit to our dose–response data with a median adjusted goodness of fit value of 0.92 (IQR 0.84, 0.94). The dose–response curves to establish drug sensitivity scores (DSS) are presented in Figure 5E [42]. No significant associations were found between DSS and molecular subtype scores (Figure 5F). We observed a trend of increasing efficacy of FLOT with increasing TME High score (Pearson *R* = 0.52) in keeping with prior results [23].

### 3.8 2‐Dimensional Cell Lines Remain a Valuable Tool to Infer Drug Effects on Certain Molecular Subtypes

Given the potential limitations of organoid culture and indiscriminate effects of cytotoxic FLOT on patient‐derived cancer organoids, we assessed whether molecular subtype scores provide insight into targeted therapy effects. Furthermore, we assessed the potential utility of existing gastric cancer cell lines to approximate molecular classification heterogeneity. Using the Cancer Cell Line Encyclopedia (CCLE) we accessed multi‐omics and drug sensitivity data for 37 gastric cancer cell lines [43].

**FIGURE 6:**
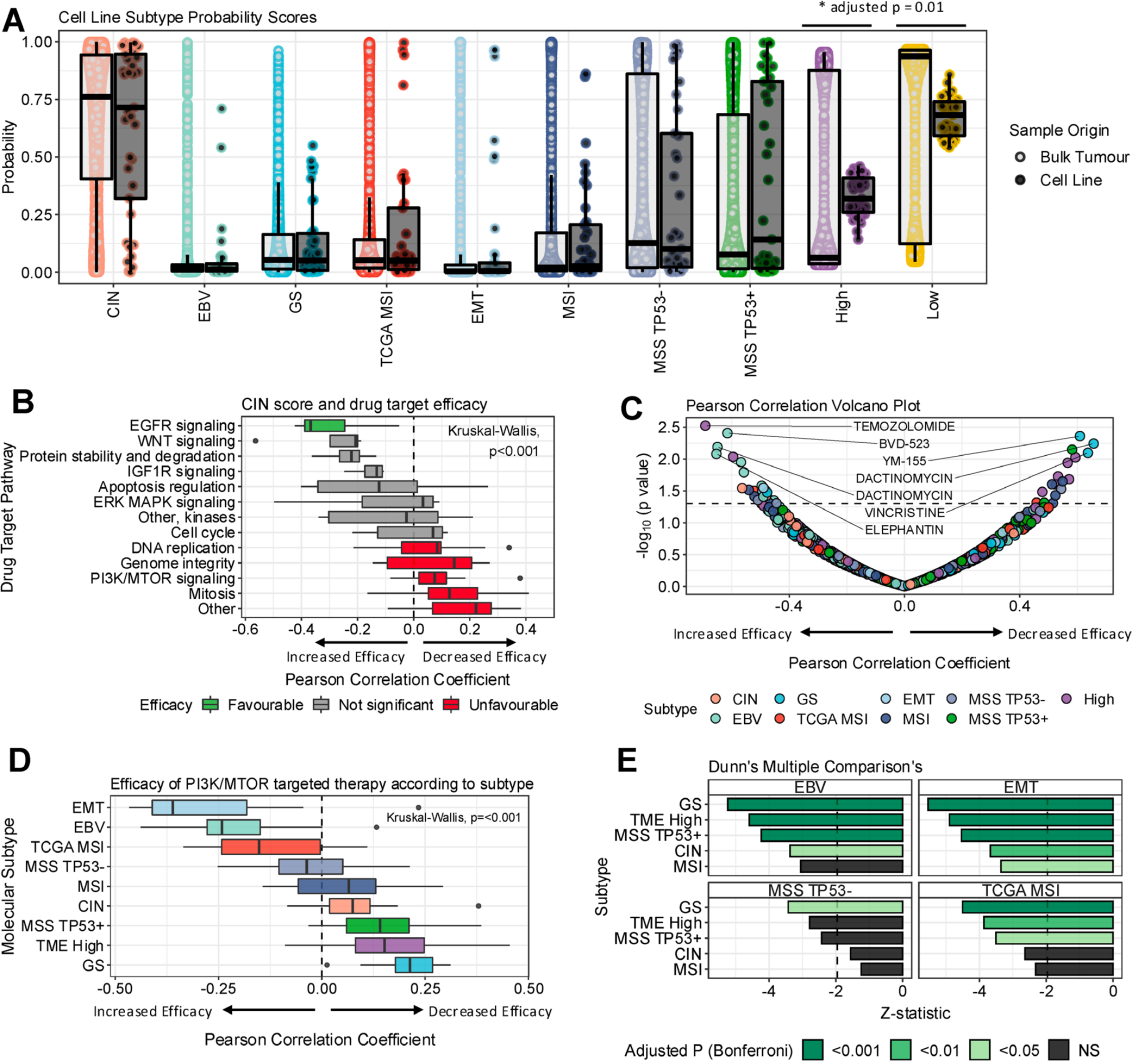
Association of subtype scores with anti‐cancer drug sensitivity. (A) Boxplot comparing the distributions of subtype scores for each molecular subtype between bulk tumour tissue and 2D cell lines. Bulk tumour distributions were determined from 2202 publicly available gastric cancer patients. Cell lines included 37 gastric cancer cell lines used in the cancer cell line encyclopedia. Bars and *p* values denote statistically significant differences using a Wilcoxon test. (B) Boxplot of drug target pathway versus Pearson's correlation coefficient for CIN subtype probability. A post hoc Dunn's test was performed. The coloured boxes identify significant favorable (green) and unfavourable (red) efficacy of drug targets in the post hoc test with Bonferroni corrected *p* values. (C) Volcano plot for univariate Pearson's correlation of molecular subtype scores and area under the curve drug sensitivity for 111 cancer drugs. The dotted line identifies the line of alpha = 0.05 for a significant unadjusted *p* value. Annotated drugs are those with the largest effect size. (D) Boxplot of molecular subtype versus Pearson's correlation coefficient for PI3K/MTOR signaling. Favorable efficacy is represented by a negative score. (E) Bar plots illustrating results of Dunn's post hoc test from plot. Statistical significance is denoted by the plot legend for subtypes that achieved any significant results.

The distribution of subtype probability scores in a cohort of 2202 gastric cancer patients was generally recapitulated among gastric cancer cell lines (Figure 6A). The only significant difference between bulk tumour sequenced molecular subtype scores and those of cancer cell lines were found between TME High and Low scores (Wilcoxon, adjusted *p* = 0.01).

Next, the efficacy of drugs against specific targeted pathways was evaluated according to molecular subtype scores. We utilized the classification system of targeted pathways proposed by the Genomics of Drug Sensitivity in Cancer. In Figure 6B we illustrate the relationship of CIN scores with targeted drugs for various pathways. We identified statistically significant efficacy of EGFR signaling targets versus targets against DNA replication, Genome integrity, PI3K/MTOR signaling, Mitosis and Other targets including RNA helicase A, NAE, IKK‐1, and IKK‐2. This is consistent with hypotheses generated by the TCGA STAD paper that highlighted the presence of receptor tyrosine kinase mutations in CIN tumours [6]. Importantly, DNA replication targets mainly consist of conventional chemotherapy agents such as epirubicin, oxaliplatin, irinotecan, and gemcitabine, among others. Given that CIN represents approximately 70% of gastric cancer cases this analysis suggests that improved survival in a large proportion of gastric cancers could be achieved by adopting targeted therapy techniques against EGFR signaling.

We investigated the in vitro effect of various anti‐cancer drugs according to each molecular subtype score at a univariate level. Using Pearson correlation, 48 drugs were found to be statistically associated with improved or decreased efficacy according to subtype score. However, after multiple comparison corrections, there were no statistically significant relationships. Amongst potential false positive comparisons, variable therapeutic targets were identified including Temozolomide, the survivin inhibitor YM‐155 and Dactinomycin (Figure 6C). Amongst the largest effect sizes, TCGA Genomically stable subtype was most commonly associated with decreased efficacy, whereas TCGA EBV was associated with improved efficacy. The results of the complete analysis are available in the Supporting Information.

In Figure 6D, we provide an alternative perspective by investigating the effect of a given target across all molecular subtypes. The complete analysis for all subtypes and targets is available in the Supporting Information. In this case, PI3K/MTOR signaling targets are significantly efficacious in EMT, EBV, TCGA MSI, and MSS TP53‐tumours relative to most other subtypes (Figure 6E). Once again, this analysis is consistent with prior studies that found TCGA EBV subtypes are enriched in PI3K mutations [6].

## 4 Conclusions

The utility of organoid models and molecular classification remains to be fully realized by the patient. Logistics and cost inhibit the widespread implementation of molecular classification, which is mainly predicated on whole‐genome measurements consisting of tens of thousands of genes [47, 48]. Furthermore, the first prospective trial evaluating whether patient‐derived colon cancer organoids could provide relevant therapeutic information to the patient failed to show feasibility [49]. To maximize the advancement of personalized medicine in gastric cancer we argue that there is a need to better understand the utility of translational pre‐clinical models and improve the cost and accessibility of molecular classification.

To enhance the clinical translation of molecular subtypes in gastric adenocarcinoma we developed and performed preliminary validation of a custom 107 gene Nanostring CodeSet using a prospective clinical cohort and patient‐derived organoids. We used gold‐standard reference tests EBV ISH and pentaplex PCR for EBV and MSI‐type tumours, respectively, to demonstrate that our test is 100% accurate in capturing these subtypes in paired tumour and tumour organoid samples. Among 38 FFPE gastric cancer specimens, our results were concordant with prior research demonstrating that diffuse‐type gastric cancer is significantly enriched in GS scores. Furthermore, we identified nearly identical proportion and molecular subtype scores for TCGA subtypes within our population compared to a cohort of 2202 gastric cancer patients. Although we found fewer TME High tumours than expected, the increased presence of TME High subtypes with MSI and EBV tumours is also concordant with other populations. These results demonstrate that our Nanostring test is accurate and feasible to perform additional study and validation.

Functional gene set analysis of the TME score associated genes identified enrichment in stromal signatures linked to cytoskeletal remodeling and cellular motility, as well as immune activation related pathways. The tRNA synthetase WARS1 and the antigen processing molecule TAP1 were identified as hub‐genes between Interferon Alpha Response and Allograft Rejection signatures. TAP1 performs the essential function of migrating peptide antigens to the endoplasmic reticulum, which facilitates antigen presentation on MHC‐1 and potentiates CD8+ activation [50]. Increased TAP1 expression in gastric adenocarcinoma has independently been demonstrated to enhance the efficacy of checkpoint inhibitor therapy [51]. Tryptophanyl‐tRNA synthetase (WARS1) performs a key role in tryptophan metabolism and protein synthesis but is also implemented in potentiating inflammatory responses in vitro and in vivo as an endogenous ligand for Toll‐like receptor (TLR) 2 and TLR‐4 [52, 53]. Increased WARS1 gene expression has been linked with improved prognosis in cutaneous melanoma and colon adenocarcinoma [54, 55].

Our network analysis also identified key bridging roles for AIF1 and FGR, suggesting potential points of interaction between stromal and immune components of the tumor microenvironment. Indeed, increased FGR has been associated with active immune signaling in nasopharyngeal carcinoma and represents a strong candidate for modulating the efficacy of immunotherapy [56, 57]. Allograft inflammatory factor 1 (AIF1) is an intriguing molecule implicated in M1‐ and M2‐macrophage differentiation [58]. Studies have identified mixed effects of AIF1 expression on tumour immune cell infiltration and prognosis [58, 59, 60]. Hub‐genes are potential key regulators of molecular functions and thus these molecules provide ideal candidates for targeted therapy aimed to modulate the tumour immune microenvironment in gastric adenocarcinoma.

The cornerstone of our study was establishing a robust methodology aimed at achieving a translational gene expression‐based test. We identified Nanostring as a desirable test due to its excellent performance on fragmented nucleic acids from formalin‐fixed paraffin‐embedded specimens [61, 62]. Nanostring also provided an efficient and affordable option to maximize clinical throughput by avoiding measurement of the whole transcriptome. High variance and high abundance genes were selected from the available transcriptomes to ensure concordance in gene counts between fresh and FFPE tissue [61, 62]. We also implemented FSQN as a cross‐platform normalization technique and novel Nanostring normalization methods with the framework proposed by Molania et al. [26, 29, 30] This method does not rely on spike‐in control probes or housekeeping genes. Instead, it assesses variation between genes of interest in the context of technical duplicates to define negative control genes that are then utilized in the Removal of Unwanted Variation (RUV)‐III algorithm. Using this framework, future gene sets derived from other models could be added to or removed from a given Nanostring assay. This plug‐and‐play approach allows optimally designed gene sets to be combined to maximize clinical utility.

One important question in organoid research is the degree to which an organoid recapitulates the molecular characteristics of the parent tumour. Numerous studies have demonstrated similarity between gene expression profiles in tumour organoid pairs [3, 63]. In this study we assessed whether this approximation of gene expression data also translates to similar molecular subtype assignments. Using our test, we identified that molecular subtype discordance between paired tumour and tumour organoids can occur, as is the case in patient 53 and 55. This result is troublesome but not surprising given the presence of intratumour heterogeneity, issues with biopsy accuracy and selective pressures exerted by tissue processing, transport and constituents of organoid media itself [64, 65, 66, 67, 68, 69]. Additional research investigating the dynamic nature of molecular subtype classes between parent tumour‐organoid pairs would enhance our understanding of potential confounding in pre‐clinical experiments.

We also assessed if more efficient and affordable 2‐dimensional cell culture can still provide a valuable pre‐clinical model. We tested the landscape of TCGA, ACRG and TME molecular subtypes, as informed by our models, in 37 gastric cancer cell lines from the CCLE [43]. With this analysis, we demonstrated that 2D cell lines are a robust method that recapitulates TCGA and ACRG classification scores but fails to generate an adequate TME High signature. Using a large drug screening assay performed by the GDSC, we also demonstrated that the strongest association between dose–response and molecular subtype scores is typically observed with molecularly targeted therapy as opposed to traditional cytotoxic chemotherapy targeting DNA replication [45]. Unfortunately, the TME signature is arguably the most important molecular subtype for prognosis and treatment in gastric cancer. Thus, cell lines may provide a prominent role in the discovery of certain therapies but more comprehensive analyses that consider the TME will require organoid, in vitro immune cell co‐culture or humanized xenograft models [70, 71, 72].

Our study contains multiple limitations. We were not able to include a “gold‐standard” reference for CIN and GS tumours. Additional validation of these subtype using whole genome sequencing is required in the future. Another limitation is that we do not have adequate follow‐up time to perform a direct comparison of dose–response to progression free survival or overall survival. Once adequate follow up time is achieved correlation of in vitro dose–response to tangible clinical outcomes should be performed.

We provide a 107 gene Nanostring assay informed by our supervised machine learning classifiers which allows molecular classification of TCGA and TME subtypes in FFPE or fresh organoid gastric cancer tissue. Our analyses suggest that a multimodal approach, which may consist of molecularly informed clinical samples, 2D, and 3D cell culture models, is required to leverage molecular classification towards personalized medicine in gastric cancer.

## Author Contributions

**D. Skubleny:** conceptualization, data curation, formal analysis, funding acquisition, investigation, methodology, software, validation, visualization, writing – original draft, writing – review and editing. **K. Purich:** data curation, investigation, writing – review and editing. **T. Williams:** conceptualization, data curation, writing – review and editing. **D. R. McLean:** data curation, formal analysis, investigation, methodology, resources, supervision. **S. N. Martins‐Filho:** data curation, formal analysis, investigation, resources. **K. Buttenschoen:** data curation, resources, writing – review and editing. **E. Haase:** data curation, resources. **M. McCall:** data curation, resources. **K. Baker:** conceptualization, methodology, resources, writing – review and editing. **S. Ghosh:** conceptualization, formal analysis, methodology, supervision, writing – review and editing. **J. L. Spratlin:** conceptualization, methodology, supervision, writing – review and editing. **D. E. Schiller:** conceptualization, data curation, funding acquisition, methodology, project administration, resources, supervision, writing – review and editing. **G. R. Rayat:** conceptualization, data curation, funding acquisition, investigation, methodology, project administration, supervision, writing – review and editing.

## Ethics Statement

All human clinical participants consented according to the approved ethics protocol granted by the Health Research Ethics Board of Alberta (Study ID: HREBA.CC‐17‐0228).

## Conflicts of Interest

D. Skubleny reports a relationship with Bold Therapeutics that includes: funding grants. G.R. Rayat reports a relationship with Bold Therapeutics that includes: funding grants. The other authors declare no conflicts of interest.

## Supporting information


**Data S1:** gcc70090‐sup‐0001‐Supinfo.docx.

## Data Availability

Data used to perform all analyses and generate plots is provided in Supporting Information at https://github.com/skubleny/Integrated‐Molecular‐Classification‐GC.
