# Supplementary material for: A 107 Gene Nanostring Assay Effectively Translates the Cancer Genome Atlas, and Tumour Microenvironment Gastric Cancer Molecular Classification to a Patient‐Derived Organoid Model
Source: Genes Chromosomes Cancer. 2025 Nov 12;64(11):e70090. doi: 10.1002/gcc.70090 (PMC12612569; doi:10.1002/gcc.70090)
Supplement: Supplementary file 1 — Data S1: gcc70090‐sup‐0001‐Supinfo.docx. [file GCC-64-e70090-s001.docx]

**Supplement**

**Contents**

1. Supplemental Methods
2. Table S1. Reagents table for human gastric cancer organoids
3. Table S2. Immunofluorescence and immunohistochemistry protocols
4. Table S3. Prospective cohort patient demographics
5. Table S4. Concordance of patient-derived organoids and parent tumour for TCGA Subtypes
6. Table S5. Concordance of patient-derived organoids and parent tumour for TME Subtypes
7. Figure S1. FLOT in-vitro drug assay validation with AGS cell line

**Supplemental Methods**

**Organoid Culture Medium**

Organoid culture media contained 1:1 basal culture medium and conditioned L-WRN cell supernatant enriched in R-spondin, noggin and Wnt (ATCC, CRL-3276). Conditioned L-cell supernatant was prepared according to Miyoshi and Stappenbeck.(1) Organoid culture media contained Advanced DMEM/F12, 2mM L-Glut, 10 mM HEPES, 100 units/mL penicillin and 100µg/mL streptomycin, 100µg/mL Primocin, 1X N2, 1X B27, 1mM N-acetylcysteine, 1nM gastrin, 10mM nicotinamide, 500nM A83-01, 10 µM SB202190 and 50ng/mL human EGF. Rho-associated kinase inhibitor (10µM Y-27632) was added for the first plating and first media change after splitting.

**Nanostring Assay**

Organoid cells (500,000 to 1,000,000 cells) were dissociated using TrypLE express and washed in PBS three times. Nucleic acids were stored at -80^o^C after cells were lysed in 350µL Buffer RLT Plus (Qiagen, 1053393) with 10µL/ml 2-mercaptoethanol and homogenized using Qiashredder tubes (Qiagen, 79654). DNA and RNA were isolated from four 10 µm Formaldehyde Fixed Paraffin-Embedded (FFPE) curls using an AllPrep FFPE DNA/RNA kit (Qiagen, 80234). The manufacturer’s protocol was followed but with the addition of proteinase-k digestion for 2 hours. Organoid DNA and RNA were separated using an AllPrep DNA/RNA kit (Qiagen, 80204). In both FFPE and organoid-derived samples, mRNA cleanup was performed using an RNA Clean and Concentrator kit (Zymo, R1015). Quality and concentration of RNA were assessed using NanoDrop 1000 (Thermo Scientific). DNA and RNA were stored at -80^o^C before downstream analysis.

***In-vitro* dose response drug assay**

AGS cells were cultured in Ham’s F-12K (Gibco), 10% FBS (Gibco) and 100 units/mL penicillin and 100µg/mL streptomycin (Gibco). Briefly, 5000 cells were plated in 96 well plates and grown for 24 hours. Anti-cancer drugs 5-fluorouracil (Tocris, 3257), oxaliplatin (Tocris, 2623) and docetaxel (Tocris, 4056) were added in triplicate over 8 half-log dilutions (5-fluororacil and oxaliplatin) or 10-fold dilutions (docetaxel). Initial concentrations for 5-fluorouracil, oxaliplatin and docetaxel were 800µM, 2400µM and 2400nM, respectively. A single 500µM dose of leucovorin (Toronto Research, L330400) was added to each treatment well. Cells were treated for 48 hours followed by a CCK-8 viability assay (Abcam, ab228554), which was performed according to the manufacturer’s protocol. We replicated our assay in three independent trials to assess reproducibility.

Nonlinear regression of dose-response data was performed using GraphPad Prism version 9. First, baseline media control absorbance was subtracted from all experimental wells. Absorbance values were then normalized between 0 and 100% given that adequate minimum and maximum treatment effect controls were present. Mean drug concentrations from 5-fluorouracil, oxaliplatin, docetaxel, and leucovorin were log10 transformed. Next, outliers were removed using Q = 1% and least squares variable slope non-linear regression estimated dose-response curves and half-maximal inhibitory concentration (IC50).

**References**

1. Miyoshi H, Stappenbeck TS. In vitro expansion and genetic modification of gastrointestinal stem cells in  spheroid culture. Nat Protoc. 2013;8:2471–82.

| Table S1. Reagents table for human gastric cancer organoids | | |
| --- | --- | --- |
| Reagent | Source | Catalog Number |
| Advanced DMEM/F12 | Gibco | 12634010 |
| L-Glut | Sigma | G8540 |
| HEPES | Fisher Scientific | BP310 |
| Penicillin Streptomycin | Gibco | 15140122 |
| Primocin | InvivoGen | Ant-pm-2 |
| N2 Supplement | Gibco | 17502011 |
| B27 Supplement | Gibco | 17504044 |
| N-acetylcysteine | Sigma | A9165 |
| Gastrin | Sigma | G9145 |
| Nicotinamide | Sigma | N0636 |
| SB202190/p 38 inhibitor | Sigma | S7067 |
| A83-01 (ALK4/5/7 inhibitor) | Sigma | SML0788 |
| Human EGF | Gibco | PHG0313 |
| Dispase II | Sigma | D4693 |
| Collagenase IX | Sigma | C9407 |
| Y-27632 | Sigma | Y0503 |

| Table S2. Immunofluorescence and immunohistochemistry protocols | | | |
| --- | --- | --- | --- |
| Protocol | Primary Antibody | Secondary antibody | Antigen Retrieval |
| 1 | Mouse Anti-pan cytokeratin, 1:25, overnight at 4 degrees Celsius, Abcam, ab7753 | Goat anti-mouse IgG Alexa Fluor 568, 1:50, 30 minutes room temperature, Invitrogen, A-11004 | Sodium Citrate |
|  | Rabbit Anti-vimentin, 1:300, overnight at 4 degrees Celsius, Abcam, ab92547 | Goat anti-rabbit IgG Alexa Fluor 488, 1:200, 30 minutes room temperature, Abcam, ab150077 | Sodium Citrate |
| 2 | Mouse anti-MUC5AC, 1:250, overnight at 4 degrees Celsius, Invitrogen, MA5-12178 | Biotinylated goat anti-mouse IgG, 1:200, 30 minutes room temperature, Jackson ImmunoResearch, 115-065-003 | Sodium Citrate |
| 3 | Rabbit Anti-pepsinogen II/PGC, 1:100, overnight at 4 degrees Celsius, Abcam, ab255826 | Biotinylated goat anti-rabbit IgG, 1:200, 30 minutes room temperature, Vector Laboratories, BA-1000 | Tris-EDTA |

| Table S3. Prospective cohort patient demographics | | |
| --- | --- | --- |
| Variable | n/N (Missing %) | N = 38^1^ |
| Age | 38 / 38 (0%) | 65 (60, 74) |
| Sex | 38 / 38 (0%) |  |
| F |  | 12 (32%) |
| M |  | 26 (68%) |
| Stage | 38 / 38 (0%) |  |
| I |  | 11 (29%) |
| II |  | 10 (26%) |
| III |  | 8 (21%) |
| IV |  | 9 (24%) |
| Grade | 38 / 38 (0%) |  |
| G1 |  | 1 (2.6%) |
| G2 |  | 10 (26%) |
| G3 |  | 26 (68%) |
| Gx |  | 1 (2.6%) |
| Tumour Location | 38 / 38 (0%) |  |
| Distal |  | 13 (34%) |
| Proximal |  | 22 (58%) |
| Whole stomach |  | 3 (7.9%) |
| Lauren Classification | 37 / 38 (2.6%) |  |
| Diffuse |  | 22 (59%) |
| Intestinal |  | 13 (35%) |
| Mixed |  | 2 (5.4%) |
| Signet Ring Cell (Positive) | 37 / 38 (2.6%) | 22 (59%) |
| ^1^Median (IQR); n (%) | | |

| Table S4. Concordance of patient-derived organoids and parent tumour for TCGA Subtypes | | | | | |
| --- | --- | --- | --- | --- | --- |
|  |  | Tumour Organoid | | | |
|  |  | CIN | EBV | GS | MSI |
| Tumour | CIN | 6 | 1 | 0 | 0 |
|  | EBV | 1 | 0 | 0 | 0 |
|  | GS | 1 | 0 | 0 | 0 |
|  | MSI | 0 | 0 | 1 | 1 |

| Table S5. Concordance of patient-derived organoids and parent tumour for TME Subtypes | | | |
| --- | --- | --- | --- |
|  |  | Tumour Organoid | |
|  |  | High | Low |
| Tumour | High | 0 | 1 |
|  | Low | 1 | 9 |


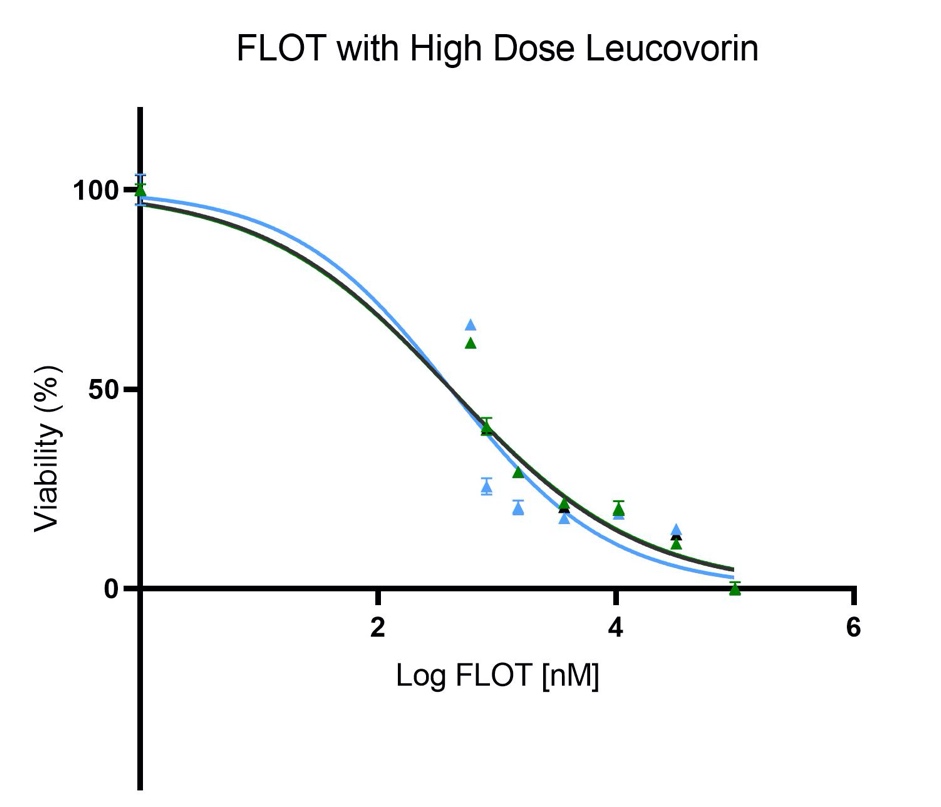


F-Test, p = 0.62

**Figure S1.** FLOT in-vitro drug assay validation with AGS cell line

. Three independent dose-response assays were performed. Cell viability was assessed using a CCK-8 assay. Variable slope least squares linear regression was used to calculate dose-response curves. A sum-of-squares F-Test demonstrated that the was best characterized by a single dose-response curve.
